# 

**Published:** 1887-01

**Authors:** 


CONTENTS.

VOL. 34 No. 1.

| | PAGE |
|---|---|
| The New Year | 1 |
| The Electric Light | 3 |
| Doctors Disagree | 4 |
| Oxygen—Its Agency in Therapeutics | 10 |
| God and the Robin | 14 |
| Electricity | 15 |
| Care of Teeth | 16 |
| How to Retain Health | 17 |
| Book Notices | 18 |
| Household | 19 |
| Miscellaneous | 21 |

INDEX TO ADVERTISEMENTS OF HALF A PAGE AND OVER.

| | PAGE. |
|---|---|
| Dwight Roberts' Hot Water Face-Bags and Throat-Bags | I |
| Lactated Food | II |
| Celerina | III |
| Lactopeptine | IV |
| Castoria | IV |
| Horsford's Acid Phosphate | V |
| Caulocorea | V |
| Crystalline Phosphate | VI |
| Bovinine | VII |
| Vaginal Injecting and Suction Syringe | VIII |
| A Bit of History | IX |
| Medicated Suppositories | XI |
| Dr. Carter Moffats' Ammoniaphone | XII |
| Goodyear Rubber Co | XII |
| Dr. Buckland's Scotch Oats Essence | 2d page cover |
| Perforated Paper | 3d " " |
| New York to Phil'a | 4th " " |

